# The Circadian-Light-Hygiene Hypothesis: A Potential Modulator of Fertility and Birthrate Trends

**DOI:** 10.3390/biology15131023

**Published:** 2026-06-26

**Authors:** Denis Gubin, Oliver Stefani, Germaine Cornelissen, Yvan Touitou

**Affiliations:** 1 Department of Biology, Tyumen Medical University, 625023 Tyumen, Russia; 2 Laboratory for Chronobiology and Chronomedicine, Research Institute of Biomedicine and Biomedical Technologies, Tyumen Medical University, 625023 Tyumen, Russia; 3 Tyumen Cardiology Research Centre, Subunit of Tomsk National Research Medical Center, Russian Academy of Science, 625026 Tyumen, Russia; 4 Department Engineering and Architecture, Lucerne University of Applied Sciences and Arts, 6048 Horw, Switzerland; oliver.stefani@hslu.ch; 5 Department of Integrated Biology and Physiology, University of Minnesota, Minneapolis, MN 55455, USA; corne001@umn.edu; 6 Unité de Chronobiologie, Fondation Ophtalmologique A. de Rothschild, 75019 Paris, France; yvan.touitou@chronobiology.fr

**Keywords:** circadian-light hygiene, light at night, melatonin, reproductive health, fertility, ovulation, circadian disruption, reproductive endocrinology, chronobiology

## Abstract

Human fertility has declined sharply since 1950, and a growing body of evidence suggests that while conventional socioeconomic factors are well-established drivers of the broader demographic transition, they do not fully account for the timing and breadth of this trend. This review examines the Circadian-Light-Hygiene hypothesis, which proposes that daily light exposure is a fundamental regulator of reproductive health. We synthesize findings from photobiology, endocrinology, reproductive medicine, and epidemiology to evaluate how artificial light at night, insufficient daytime light, and irregular light–dark patterns may disrupt the hormonal timing systems that support reproduction. The available evidence indicates that such disruption can alter melatonin signaling, circadian gene regulation, and neuroendocrine rhythms, with downstream effects on ovulation, sperm quality, endometriosis, polycystic ovary syndrome, pregnancy outcomes, and developmental programming. Urbanization, screen use, and shift work appear to amplify these effects, while genetic variation may modify individual susceptibility. Although direct causal evidence in humans remains limited for several endpoints, the convergence of observational, experimental, and translational data supports circadian-light misalignment as a plausible, additional modulator of fertility decline, and a potentially modifiable contributor. Optimizing daily light exposure may therefore represent a low-cost and scalable strategy for improving reproductive health.

## Simple Summary

Human fertility has been declining worldwide for decades, and many countries now have birth rates below the level needed to replace their populations. Traditional explanations such as social change, contraception, and later childbearing do not fully explain why this decline is happening so widely and so quickly. In this review, we propose that the way people are exposed to light every day may be an important but overlooked factor. We summarize evidence showing that too much light at night, too little daylight, and irregular daily light patterns can disrupt the body’s internal timing system, which helps control hormones involved in reproduction. These changes may affect ovulation, sperm quality, pregnancy, and fetal health. We also discuss how modern city life, screen use, and shift work may worsen this problem. Understanding how daily light affects reproductive health could lead to simple, low-cost ways to support fertility, such as better lighting design, healthier sleep schedules, and timed light exposure.

## 1. Introduction

Global fertility rates have undergone a dramatic decline, halving since 1950, with over 55% of countries now falling below replacement level. While conventional socioeconomic and demographic models offer valuable insights, they do not fully account for the timing, pervasive geographic consistency, and rapid acceleration of this unprecedented fertility crisis [1,2]. This review posits that Circadian-Light Hygiene (CLH), defined as the alignment of daily light exposure with natural diurnal and seasonal cycles, represents a crucial, yet underappreciated, biological contributor to contemporary fertility loss alongside established socio-economic drivers.

We propose that CLH disruption, encompassing excessive artificial light at night (LAN), insufficient daytime illumination, and irregular light–dark patterns [3,4,5], acts as a systemic contributing factor that dysregulates the hypothalamic–pituitary–gonadal (HPG) axis. This dysregulation is primarily mediated by the suppression of melatonin, the perturbation of core clock gene expression, and the desynchronization of critical neuroendocrine rhythms. The downstream consequences manifest as a spectrum of reproductive dysfunctions, including impaired ovulation, polycystic ovary syndrome (PCOS), endometriosis, diminished sperm quality, and the metabolic programming of offspring. Our objective in this review is threefold: first, to synthesize and critically evaluate the empirical evidence linking CLH disruption to reproductive dysfunction across sexes and diverse life stages; second, to identify urbanization and educational attainment as significant amplifiers of this exogenous biological stressor within the broader demographic transition; and third, to propose actionable, low-cost, and scalable interventions aimed at restoring circadian alignment and thereby ameliorating fertility outcomes. CLH constitutes a unifying framework for 24 h light health, defined by the alignment of light exposure with natural diurnal and seasonal cycles that facilitates robust circadian entrainment [6,7,8,9]. Maintaining optimal CLH requires the mitigation of three fundamental stressors: (1) excessive artificial LAN [10], (2) insufficient daytime light exposure [11], and (3) irregular light–dark periodicity [3,12,13]. The physiological basis of CLH relies largely on melanopic stimulation, quantified using melanopic Equivalent Daylight Illuminance (m-EDI) in accordance with CIE S 026/E:2018.; CIE System for Metrology of Optical Radiation for ipRGC-Influenced Responses to Light. International Commission on Illumination: Vienna, Austria, 2018 [14]. This pathway, characterized by melanopsin’s peak sensitivity near ~480 nm [15] or around 490 nm when prereceptoral filtering is considered, primarily facilitates melatonin suppression [14,16] and circadian phase entrainment [16] through intrinsically photosensitive retinal ganglion cells (ipRGCs). Given that static indoor illumination remains the default in modern environments, there is a critical need to transition toward adaptive, spectrally dynamic lighting systems [17,18] that emulate the natural daylight range to preserve circadian health in urbanized populations.

Beyond circadian disruption, modern lifestyle alters the ensemble of environmental time cues that historically synchronized reproductive physiology, including circatrigintan/circalunar (~29.5-day) and circannual (~yearly) rhythms—all modulated by artificial light and noise signals. Circannual rhythms robustly govern seasonal breeding across vertebrates: birds [19,20], mammals including rodents and rams [21,22], and humans show seasonal fertility/birth patterns and hormone seasonality [23,24,25]. While circalunar reproductive rhythms are well-documented in marine fish and invertebrates [26], evidence in humans is limited (menstrual–lunar synchronization remains controversial) [27]. In canids, reproductive seasonality varies markedly by species and housing status; the domestic dog is generally less seasonal than many wild canids, but the family as a whole retains clear photoperiod-sensitive reproductive diversity, underscoring the importance of ancestral timing cues in the face of modern environmental disruption [28]. This broader mismatch with the ancestral evolutionary template, not merely altered light exposure alone, may underlie the decline in fertility.

Whether arising from environmental photoperiodic extremes or anthropogenic light exposure, disruptions to CLH dampen reproductive endocrine rhythms through shared physiological mechanisms. For instance, high-latitude populations experience seasonal CLH volatility: winters marked by light deficits in the morning and summers by light excess in the evening. These extremes flatten the circadian amplitude of melatonin and delay its phase [29]. They correlate with adverse metabolic markers such as elevated TC/HDL-C and TG/HDL-C ratios [30,31], which are established predictors of infertility. In contemporary societies, these natural photoperiodic challenges are compounded by pervasive artificial LAN [10,32,33,34,35,36] and sedentary, urban-centric lifestyles. Importantly, spring and summer photoperiods are stimulatory to the HPG axis in humans (e.g., higher ovulation rates, improved IVF outcomes), while the inhibitory effects arise from altered circadian amplitude and timing, specifically flattened amplitude and phase delay of melatonin, rather than from changes in photoperiod length [29,30].

**Figure 1:**
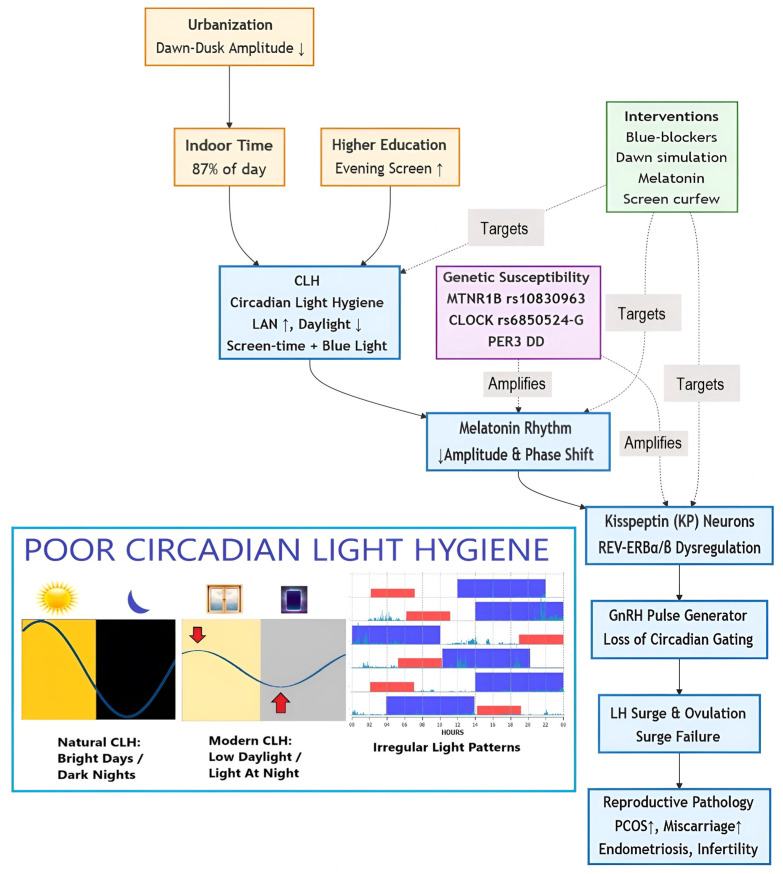
Circadian-Light-Hygiene Disruption: Mechanistic Pathway to Reproductive Dysfunction and Proposed Interventions. This flowchart depicts how poor CLH (↑ light-at-night, ↓ daylight amplitude, blue light excess) dysregulates melatonin rhythms, REV-ERBα/β, kisspeptin signaling, and GnRH pulsatility, culminating in LH surge failure, ovulation defects, and pathologies (PCOS, miscarriage, endometriosis). Urbanization (87% indoor time), higher education (evening screens), and genetic variants (MTNR1B rs10830963, CLOCK rs6850524, PER3^DD) amplify effects; interventions (blue-blockers, dawn simulation, melatonin, screen curfews) target early nodes. “Irregular Light Patterns” illustrate real actigraphy data showing high inter-daily variability in light exposure, quantified as onset/offset times of the 10 h maximum (blue) and 5 h minimum (red) irradiance periods. Large day-to-day variance indicates unstable circadian synchronizing strength, characteristic of modern light hygiene disruption.

Anthropogenic factors profoundly disrupt CLH. The pervasive use of blue-enriched LED screens and prolonged indoor work environments artificially extend evening light exposure, mimicking the summer solstice photoperiod year-round. Shift work represents an extreme manifestation of circadian misalignment, compelling individuals to engage in activity and light exposure during biological night [37,38]. Behaviorally, the high prevalence of late chronotypes and social jet lag (SJL), the discrepancy between social and biological time, establishes a chronic state of circadian disruption [39,40,41]. Urban design further exacerbates these challenges; limited access to natural green spaces and high-rise residential settings frequently curtail exposure to the intense, timing-critical morning daylight essential for robust circadian entrainment [42,43,44]. To provide a structured overview of these interactions, the comprehensive CLH framework and its multi-level impacts on fertility are synthesized in Figure 1.

Importantly, the CLH hypothesis operates through shared physiological mechanisms in both sexes. While the review includes substantial coverage of female reproductive endpoints (ovarian function, PCOS, endometriosis, pregnancy outcomes, and maternal–fetal programming), Section 5 presents parallel evidence for male fertility, including sperm quality, testicular clock-gene expression, and oxidative stress. Together, these findings demonstrate that CLH is a systemic, sex-neutral biological stressor affecting the full spectrum of human reproduction.

This review’s mechanistic evidence stems primarily from nocturnal rodent models, necessitating caution when applying findings to diurnal humans. Although data from diurnal animals are included, such studies are limited. Fortunately, the growing body of human evidence from actigraphy and wearables, which allows the direct assessment of circadian-light disruption, reduces reliance on results from animal studies. The absence of diurnal/crepuscular animal models mirroring human circadian patterns is a key area for future investigation.

Search Strategy and Scope: This narrative synthesis retrieved literature from PubMed/MEDLINE, Web of Science, and Scopus (primary databases), with Google Scholar for supplementary searching. The review primarily covers publications from 2005–2025 (last 20 years), reflecting the rapid expansion of circadian-light-hygiene (CLH) research. Fundamental earlier work was included when mechanistically pertinent. Citation selection reflects expert identification of mechanistically relevant studies rather than systematic screening, appropriate for narrative synthesis methodology.

## 2. Educational and Urban Determinants of the Dampened Circadian Amplitude of Light Exposure

Urbanization and higher education are two of the most robust predictors of declining fertility rates globally [45]. Yet, their biological mechanisms remain inadequately explained by socioeconomic models alone. Emerging evidence supports the CLH hypothesis: the suppression of reproductive potential in modern societies is not merely a cultural or economic choice, but partly a physiological consequence of disrupted light–dark cycles that impair HPG axis function in conjunction with established socio-economic factors [46]. Urban women exhibit fertility rates 11–15% lower than rural women, even after controlling for age, income, and education, suggesting that urbanization itself acts as an independent biological suppressor of fertility that amplifies existing demographic trends [47]. This “urban residual” is a state of circadian amplitude suppression, where chronic daylight deficiency and nocturnal light exposure create a “biological twilight” that fails to adequately trigger the HPG axis, particularly in genetically susceptible individuals [48]. Modern urban residents spend 87% of their time indoors, where ambient light levels are reduced by up to 10-fold compared to natural daylight [6,49,50]. This chronic light deprivation blunts the amplitude of the circadian rhythm, weakening the dawn-to-dusk signal that synchronizes GnRH pulses and optimizes ovarian and testicular function (see Table 1).

Table 2 shows a strong inverse correlation between the percentage of a nation’s population living under pristine night skies (minimal LAN) [51] and the projected 2025 Total Fertility Rate (TFR) [52]. High-pristine countries such as Chad (78.7% pristine, TFR 5.94) and Somalia (74.4% pristine, TFR 5.91) have markedly higher fertility than highly urbanized nations with near-zero pristine sky exposure. South Korea, for example, has 0% pristine sky and a TFR of only 0.75. Across the dataset, pristine population percentage and TFR show a robust negative correlation (r ≈ −0.85). This finding supports the CLH hypothesis: low LAN exposure is associated with maintained reproductive function amid a global fertility decline. Argentina (1.4% pristine, TFR 1.51) is an exception. It aligns with Tassino & Leone (2025) [53], who report that the Río de la Plata region shows pervasive eveningness, sleep deficits, and severe social-biological misalignment. These factors likely override the benefits of relatively lower LAN. Pakistan (3.4% pristine, TFR 3.50) and Israel are cultural outliers. Pro-natalist norms partially counteract biological pressures. Nevertheless, their TFRs are still lower than those of sub-Saharan nations with comparable LAN exposure, indicating that CLH effects remain detectable even under cultural moderation.

Higher education amplifies the effect. Academic environments systematically violate light hygiene. Students and professionals spend 5 to 8 h per day in front of LED-backlit screens, often during evening hours. Such screens emit intense blue-enriched light (450–480 nm) that potently suppresses melatonin via melanopsin photoreceptors [54,55,56,57]. A single evening of screen use delays circadian phase by about 1.5 h, increases sleep latency, and reduces nocturnal melatonin by up to 50% [56,57,58]. This “Zoom-Peak” phenomenon imposes artificial midday-like illumination during biological night. It creates “circadian poverty”: a flattened diurnal light amplitude that the hypothalamus interprets as environmental instability, thereby down-regulating reproductive investment. Quantitative studies show that achieving at least lower secondary education and high modern contraceptive prevalence are the strongest predictors of fertility decline [59]. Education shapes daily schedules and technology use, promoting later bedtimes and more evening screen exposure that affect circadian rhythms [60]. These habits reduce natural daylight and increase blue-enriched LAN. The result is altered melatonin secretion with delayed peaks and flattened profiles [61]. This pathway links educational attainment and family-planning policies to reproductive outcomes within the broader demographic transition. Elevated evening cortisol, driven by chronic light disruption and poor sleep, further dysregulates the HPG axis. It impairs ovulation, sperm quality, and embryo implantation [61,62].

Individuals exhibiting a later chronotype are particularly vulnerable to circadian disruption. They possess an inherent predisposition toward evening activity, a propensity that is further exaggerated by screen use, which leads to heightened melatonin suppression and disrupted sleep architecture [63], including sleep duration [57]. Educated urban populations disproportionately comprise late chronotypes, likely because academic and professional environments favor evening schedules. This alignment of social expectations with biological preference amplifies circadian misalignment precisely when reproductive physiology is most sensitive to timing. Consequently, the impact extends beyond the well-documented trend of delayed childbearing [64,65]. There is emerging evidence for a physiological suppression of fertility potential, even during active attempts at conception, which is most pronounced among highly educated cohorts [66,67]. Within these groups, higher educational attainment correlates significantly with systemic circadian misalignment [68]. Even in instances where completed fertility remains unchanged, the physiological readiness for conception is often compromised. The scale of this misalignment is underscored by the high prevalence of social jetlag (SJL) among university students [69]. This phenomenon is largely dictated by social commitments; indeed, SJL is notably attenuated under relaxed social schedules, such as those observed during COVID-19 lockdowns [70,71]. Furthermore, when adequate outdoor light exposure is maintained, the negative impacts of these social schedules on circadian markers can be partially mitigated [72]. Therefore, the evidence suggests that education does not merely delay parenthood, it actively reprograms the circadian architecture essential for reproduction.

Re-framed through the CLH lens, the fertility decline reflects an evolutionary mismatch. Our reproductive physiology was calibrated by millennia of predictable light–dark cycles. It now operates under artificial, attenuated, and phase-delayed photic environments. Urbanization and advanced education thus function as unintentional environmental endocrine disruptors that contribute to established socio-economic drivers of fertility decline. Their most insidious “toxin” is not chemical; it is the absence of accurately timed dawn signals. People with genetic variants that confer heightened light sensitivity, such as polymorphisms in MTNR1B, or those who acquire circadian vulnerability through conditions like post-COVID hypothalamic–pineal disruption [73,74,75] are at disproportionately high risk of fertility suppression under modern light environments. These individuals serve as biological sentinels of the Circadian-Light Hypothesis.

**Table 1:** Determinants of Circadian-Light-Hormone Disruption and Their Reproductive Consequences.

Determinant	Evidence & Effect	Evidence & Effect	Reproductive Impact
Urbanization	11–15% lower fertility in urban vs. rural women [47] → chronic daylight deficiency [9] + Light at Night	87% indoor time [49] → 10-fold lower ambient light → blunted dawn-to-dusk amplitude → weakened circadian entrainment [9]	↓ GnRH pulsatility → reduced LH surge → anovulation, miscarriage
Higher Education	Less daylight + increased evening screen time → later chronotype; delayed first birth	LED backlit screens (450–480 nm) suppress melatonin ~50%; phase delay ~1.5 h [55,56]	Suppressed nocturnal melatonin + elevated cortisol → impaired ovulation, sperm quality, embryo implantation
Indoor Time	87% of day spent indoors [49]	Reduced natural daylight → smaller amplitude of circadian rhythm [9]	Same as urbanization
Screen time & Blue-enriched Light	5–8 h/day exposure in students/professionals.	Melanopsin activation → melatonin suppression, cortisol rise	Same as higher education
Social Jet Lag	82% prevalence among university students [59]	Misalignment of social and biological time → desynchronization of kisspeptin–GnRH and melatonin-GnRH axis	↓ fertility motivation, delayed conception
Genetic Susceptibility	*MTNR1B* rs10830963 [76,77,78,79,80], *CLOCK* rs6850524 G, rs11932595 G [81,82], *PER3 DD* [83], *REV-ERBα/β* [84,85,86,87,88,89] variants.	Modulate sensitivity to melatonin suppression and circadian amplitude	Individuals with these variants experience amplified fertility suppression under CLH disruption
Interventions	Light hygiene, daylight exposure, melatonin-rich food, chronotherapy	Restore circadian amplitude & phase, re-entrain reproductive axis	Potential reversal of CLH-related fertility decline

Note: Urban living and higher education amplify Circadian-Light-Hygiene disruption through increased indoor confinement and evening blue light exposure. These environmental factors, together with genetic predispositions, dampen circadian amplitude, suppress melatonin, and desynchronize the reproductive hormonal cascade, leading to measurable declines in fertility. Targeted CLH interventions can restore synchrony and mitigate these effects.

**Table 2:** Population Under Pristine Night Skies Versus Total Fertility Rate: LAN (Light at Night) and TFR (Total Fertility Rate) Correlation.

Country	Pristine Pop %, 2016	TFR, 2025 UN
Chad	78.7	5.94
Somalia	74.4	5.91
Central African Republic	78.2	5.81
Niger	73.2	5.79
Madagascar	76.7	3.85
Pakistan	3.4	3.50
Israel	0.0	2.75
Saudi Arabia	0.0	2.29
Libya	0.9	2.25
Qatar	0.0	1.70
Argentina	1.4	1.51
Kuwait	0.0	1.50
United Arab Emirates	0.0	1.21
Singapore	0.0	0.96
South Korea	0.0	0.75

Notes: Data from Falchi et al. (2016) [51] New World Atlas (population % under pristine skies, <1% artificial brightness; ‘pristine” skies have zenith luminance ≤ 0.01 times natural dark-sky background, i.e., ~0.067 mcd/m^2^ artificial addition) and UN World Population Prospects 2024 (TFR, births per woman in 2025) [52]. High pristine % inversely correlates with TFR (r ≈ −0.85). Sorted by descending TFR. TFR denotes Total Fertility Rate (average births per woman). LAN is Light at Night (artificial nocturnal illumination). Pristine Pop % serves as a proxy for minimal LAN exposure using VIIRS-DNB (Visible Infrared Imaging Radiometer Suite Day/Night Band) satellite radiance data (calibrated via 30,000+ citizen sky measurements) and population grids, the atlas models skyglow per 0.25° × 0.25° cell. Pristine Pop % sums population in cells below this threshold, divided by national total—indicating unpolluted rural/low-density areas resilient to circadian disruption. This association likely co-varies with broader socioeconomic development, urbanization, and the demographic transition, and cannot be used alone to infer individual-level mechanisms. The correlation serves as population-level evidence supporting the CLH hypothesis, but causal inference at the individual level requires longitudinal, mechanistic studies.

### 2.1. The Light-Hormone Axis: Circadian Regulation of Reproductive Physiology

**Figure 2:**
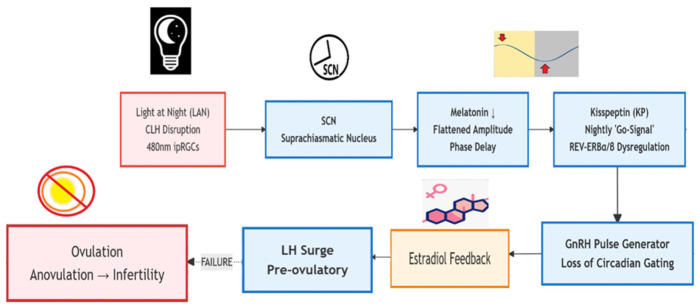
Light at night (LAN) suppresses melatonin and alters its circadian rhythm (amplitude and phase), disrupting the nightly KP “go-signal” from the SCN that gates the GnRH pulse generator. Loss of circadian synchronization prevents the estradiol-triggered LH surge required for ovulation, leading to anovulatory infertility.

For billions of years before electric light existed, almost every organism evolved under strict day–night alternations, internalizing Earth’s about 24-h spin into self-sustaining circadian clocks; alignment of these molecular clocks with environmental light–dark cycles is therefore particularly critical for reproductive physiology [6,48]. CLH disruption may impair fertility in part by altering melatonin rhythms and thereby affecting the timing of reproductive hormone signaling. At the same time, current evidence indicates that this mechanism is incomplete on its own, since ovulatory failure likely arises from interactions among melatonin, central circadian pathways, and other neuroendocrine regulators. Specifically, CLH disruptions flatten the circadian amplitude of melatonin and delay its phase. These shifts decouple the suprachiasmatic nucleus (SCN) from the kisspeptin (KP)—gonadotropin-releasing hormone (GnRH) network—the master gate that times the pre-ovulatory luteinizing-hormone (LH) surge (Figure 2).

Loss of this nightly KP “go-signal” is a direct route to anovulatory cycles [90,91]. Melatonin alterations also interfere with the HPG axis because the LH surge necessary for ovulation is tightly coupled to circadian signals from the SCN, and its timing is influenced by cortisol rhythms and estradiol feedback [92,93]. A diminished or mistimed SCN output, as occurs with small-amplitude or delayed melatonin rhythms, can disrupt this synchronization, leading to irregular ovulation and impaired fertility [94,95].

Melatonin exerts profound direct effects on ovarian function [96,97,98], extending beyond its classical role as a circadian and photoperiodic regulator [99,100]. Recent research further illuminates the complexity of melatonin regulation, demonstrating that its suppression by light is significantly influenced by an individual’s sex and the season, with females and winter conditions showing heightened sensitivity [101]. This study also highlighted an important menstrual cycle phase dependency, finding that females during their luteal phase had earlier Dim Light Melatonin Onset (DLMO) than those in their follicular phase, underscoring the dynamic nature of circadian timing in women [101]. Acting through melatonin receptors, including MTNR1A and MTNR1B [99,102,103,104,105,106,107,108,109,110,111,112,113,114,115,116,117,118] and via its potent, non-receptor-mediated antioxidant “cascade” [99,118,119], melatonin directly influences key ovarian processes. It promotes follicle growth [106] and oocyte quality [100,107,108] by reducing oxidative stress and apoptosis in granulosa [109,110,111,112,113] and cumulus cells [114,115], thereby slowing follicular atresia. In the corpus luteum, melatonin modulates progesterone synthesis, often stimulating it through pathways like Adenosine Monophosphate activated Protein kinase/Mechanistic Target of Rapamycin (AMPK/mTOR) [116], while protecting luteal cells from oxidative damage [116,117,118], thus supporting luteal function and pregnancy [97,108,119,120]. Furthermore, melatonin enhances outcomes in assisted reproduction, including ovarian tissue cryopreservation [121] and embryo implantation [96,122]. These multifaceted actions highlight melatonin’s critical role as an endogenous regulator of ovarian physiology and its potential therapeutic application in fertility preservation and treatment.

Clinically, interventions targeting melatonin pathways have been tested in humans with measurable impacts on fertility outcomes. Oral melatonin supplementation (3–10 mg/day) consistently improves oocyte quality, fertilization rates, and pregnancy outcomes in women with diminished ovarian reserve or PCOS, as demonstrated in multiple randomized controlled trials (reviewed in [123]). Specifically, melatonin supplementation has been tested in humans for various fertility-related conditions, demonstrating efficacy in improving oocyte quality, uterine receptivity, and pregnancy outcomes. Clinical studies have evaluated its use in women with poor oocyte quality undergoing intracytoplasmic sperm injection (ICSI), showing increased fertilization rates, mature oocytes, and high-quality embryos [124,125,126,127,128,129,130,131,132,133,134,135,136,137,138,139,140,141]. Additionally, melatonin has been tested in women with PCOS, enhancing oocyte and embryo quality [128], and in cases of endometriosis, reducing pain and dysmenorrhea [129]. Its benefits extend to repeated implantation failure (RIF) and pregnancy loss, where it modulates immune responses to improve uterine receptivity [130]. Furthermore, melatonin has been investigated for its role in placental health, alleviating oxidative stress and improving vascular dynamics [131]. These interventions highlight melatonin’s potential as a therapeutic agent across multiple dimensions of female infertility.

Non-pharmacological approaches, such as morning bright light therapy (e.g., 10,000 lux for 30–60 min), have also been shown to modulate the hypothalamic–pituitary–ovarian axis, increasing gonadotropin secretion and follicular development, effects that appear partially independent of melatonin suppression [94,132]. Regarding receptor signaling, increased daytime melatonin, whether due to shift work, artificial light exposure at night, or exogenous supplementation, can desensitize melatonin receptors (particularly MTNR1B) through chronic overstimulation, potentially blunting the amplitude of the nocturnal melatonin peak. This phenomenon may mimic a state of functional nocturnal melatonin deficiency, impairing receptor-mediated signaling in ovarian tissues despite elevated total daily melatonin exposure. Thus, the timing and rhythmicity of melatonin secretion, not merely its concentration, are critical for optimal reproductive signaling.

Overall, circadian disruptions mediated by the light-hormone axis carry profound implications for population-level fertility and birthrate management. Anovulatory cycles from SCN-KP desynchronization directly contribute to rising infertility rates, particularly among urban young women exposed to chronic LAN and screen light, Figure 2. The loss of melatonin’s oocyte-protective and luteal-supporting functions compounds this effect, accelerating reproductive aging and reducing natural conception success.

### 2.2. Seasonal and Latitude Features

In high-latitude regions, CLH is profoundly disrupted by extreme photoperiods. Longitudinal Arctic studies have demonstrated that equinoxes provide the most stable CLH conditions, whereas both winter and summer solstices induce circadian misalignment, delaying melatonin and affecting morning cortisol and lipids through prolonged darkness and low natural daylight in winter and through excessive evening light exposure in summer [29,30,31]. While these studies do not directly assess fertility, such chronic circadian disruption is biologically plausible as a modulator of the hypothalamic–pituitary–ovarian (HPO) axis, potentially delaying ovulatory timing or reducing conception probably during periods of extreme photoperiod stress. Large-scale demographic data reveal a consistent latitudinal gradient in human birth seasonality [133,134,135]. Martinez-Bakker et al. (2014) [136] analyzed U.S. birth records (latitude range: ~25–49° N) over 78 years, identifying peak births in late spring/early summer in northern states and autumn peaks in southern states. These patterns persisted after adjusting for socio-cultural factors like holidays and weekends, suggesting underlying biological drivers. This pattern suggests that reproductive timing is evolutionarily tuned to regional environmental constraints, with high-latitude populations favoring births during optimal thermal and nutritional windows, and moderate-latitude populations influenced more strongly by socio-cultural factors such as holiday-driven conception surges. In moderate-latitude populations, where photoperiodic extremes are absent, reproductive timing appears less governed by photoperiod and more by behavioral and seasonal lifestyle shifts. Autumn birth peaks correlate with increased sexual activity during winter holidays [137,138]. Clinically, in vitro fertilization, IVF outcomes show modest but significant seasonal variation, with higher clinical pregnancy rates observed in spring and summer, particularly under long protocol cycles and within a narrow ambient temperature range (26.13–29.68 °C), though these effects are protocol-dependent and not universally replicated [139]. These findings may reflect multiple interacting factors; direct circadian effects cannot be definitively disentangled from temperature or socio-cultural rhythms. At moderate latitudes, CLH likely plays a permissive rather than directive role, with metabolic and thermal stability potentially enhancing endometrial receptivity alongside circadian effects.

Emerging evidence links CLH directly to ovarian physiology. Anti-Müllerian hormone (AMH), a biomarker of ovarian reserve synthesized by granulosa cells, exhibits weak but statistically significant seasonal variation in women aged 30–40, with modest peaks in spring and autumn correlating with moderate solar radiation [140]. This relation may reflect photoperiodic modulation of follicular recruitment, though confounders such as vitamin D status and activity patterns remain inadequately controlled. Experimental studies provide further mechanistic support: morning exposure to bright artificial light (4300 lux) during the follicular phase significantly increases serum LH, FSH, and prolactin, enhances follicular growth, and improves ovulation rates in women with oligo-ovulation [132]. Population-level data from over 100,000 women in Sweden and the U.S. showed that longer daylengths correlate with higher ovulation rates and increased self-reported sexual behavior, even after adjusting for temperature and social activity [141].

Overall, the convergence of demographic, clinical, and experimental data suggests that CLH operates as a modulatory layer on reproductive physiology, fine-tuning ovulatory timing and conception likelihood in response to photoperiodic cues, particularly at high latitudes. At high latitudes, disruption related to extreme photoperiods may directly impair HPO axis function; at moderate latitudes, CLH acts permissively alongside metabolic and thermal factors, contributing to fertility decline as part of a broader set of interacting factors rather than as a standalone causal mechanism. However, the precise neural and endocrine pathways linking CLH to the HPO axis, and how they interact with genetic, cultural, and metabolic variables, remain poorly understood. Future research should address longitudinal, photoperiod-controlled studies across latitudes, integrating actigraphy, melatonin profiling, and ovarian biomarkers to isolate CLH’s independent effects on human fertility.

### 2.3. Circadian Disruption and Polycystic Ovary Syndrome (PCOS)

PCOS affects up to 20% of reproductive-age women [142] and is recognized as the most common cause of anovulatory infertility [143]. The CLH hypothesis posits that chronic misalignment between environmental light exposure and endogenous circadian rhythms drives PCOS through melatonin suppression, clock gene dysregulation, mitochondrial dysfunction, and androgen excess, thereby impairing ovarian function and fertility. It necessitates a paradigm shift in reproductive medicine toward circadian-informed prevention and chronotherapeutic interventions as a foundational strategy for managing PCOS and restoring birthrate potential. Studies hinting at a temporal dimension to the disorder include associations between shift work and PCOS (n = 436 PCOS and 715 controls) [144], between circadian disruption and PCOS (n = 841 from 9 studies) [145] (reviewed in [146,147]), and evidence for involvement of clock genes [148,149,150]. Subsequent studies have transformed this correlation into causal inference. Using Mendelian randomization in over 330,000 individuals of European ancestry, Dilimulati et al. (2025) [149] demonstrated that a genetic predisposition to earlier wake times and morning chronotype confers a reduced risk of PCOS, independently of BMI. This research also found that a shift in the mid-sleep point to a time after 04:00 a.m. is associated with elevated total testosterone, suggesting that the circadian phase should be considered as an independent PCOS determinant rather than a concomitant symptom. However, targeted chronobiological studies are needed to assess the phase of the circadian rhythm in testosterone, which likely influences testosterone measured at a fixed time in the morning.

Profound dysregulation of core circadian clock genes (CLOCK, BMAL1, NPAS2, PER1/2, CRY1/2, DEC1/2, NR1D1, and RORA) in peripheral blood mononuclear cells of PCOS patients was recently identified [84]. Crucially, they demonstrated that CLOCK (circadian locomotor output cycles kaput, core clock transcription factor)/BMAL1 (brain and muscle ARNT-like 1, clock co-activator) knockdown directly upregulates SRD5A1/2 (steroid 5-alpha-reductase types 1 and 2, DHT-synthesizing enzymes) and CYP17A1 (cytochrome P450 17A1, androgen-producing enzyme), increasing dihydrotestosterone (DHT) production while suppressing estradiol synthesis. This effect provides a direct molecular link between circadian gene disruption and androgen excess, a cardinal feature of PCOS. The observation that these same genes are altered in DHEA (dehydroepiandrosterone, adrenal androgen precursor)-induced PCOS mice confirms a conserved, tissue-independent mechanism. A recent study of 16 women with PCOS and 12 healthy controls showed that RNA-sequencing and quantitative polymerase-chain-reaction (qPCR) analyses revealed significantly elevated and rhythm-disrupted Period 3 (PER3) gene expression in PCOS granulosa cells, accompanied by reduced follicular melatonin and estrogen [150].

Multiple lines of evidence converge on melatonin dysregulation as a crucial PCOS mechanism [98]. Lower melatonin concentrations in follicular fluid of PCOS women undergoing IVF were found to correlate with reduced high-quality embryo rates [151]. This association suggests a local ovarian microenvironment deficit, impairing oocyte maturation and redox balance. Phase delay and amplitude dampening of melatonin can, once again, be involved in combination with compromised metabolism. Simon et al. (2019) [152] found that obese adolescent girls with PCOS exhibit a later melatonin offset, meaning melatonin secretion persists into the morning hours, a proxy of circadian misalignment. As reported by Terzieva et al. (2013) [153], this delayed melatonin offset is compounded by elevated daytime melatonin, indicative of a blunted 24-h rhythm. Heydari & Ramdass (2025) [154] provided a meta-analytic confirmation using pooled data from 1100 women, confirming a mean melatonin difference of +14.3 pg/mL in PCOS, primarily due to elevated daytime values, alongside reduced sleep efficiency and elevated evening cortisol. This paradoxically elevated daytime melatonin yet reduced ovarian melatonin suggests that the timing or signaling of melatonin secretion is impaired, rather than that melatonin is simply overproduced. Certain genes such as MTNR1B rs10830963 are known to be linked to a delayed melatonin offset [155] or phase, specifically under compromised light hygiene [156,157]. The MTNR1B rs10830963 and MTNR1A rs2119882 polymorphisms are strongly associated with PCOS risk [76,77,78,79], further implicating melatonin receptor dysfunction. Furthermore, these SNPs are linked to insulin resistance, hyperglycemia, and elevated LH/testosterone, mirroring the metabolic-reproductive phenotype of PCOS [78,80,158]. Critically, LAN exposure may potentiate these genetic risks, creating a “double-hit” model: genetically susceptible individuals exposed to chronic LAN exhibit amplified circadian and metabolic disruption.

CLH posits outdoor and indoor LAN as a primary environmental endocrine disruptor. Using high-resolution satellite imagery and a cohort of 20,633 women, a dose-dependent, nonlinear association between LAN exposure and PCOS prevalence (OR = 1.4 for highest quintile) was demonstrated [159]. Higher LAN correlated with lower ratio of follicle-stimulating hormone to luteinizing hormone (FSH/LH), elevated testosterone, insulin resistance, and impaired glucose homeostasis, a profile consistent with ovarian hyperandrogenism and metabolic dysfunction. Notably, the effect was strongest in younger, obese, and rural women, populations with higher susceptibility to circadian disruption. Given that this effect was stronger in younger women, adolescent LAN exposure may constitute a potential developmental risk factor for adult PCOS. This finding is mechanistically aligned with Zhou et al. (2025) [160], who showed that bedroom LAN exposure accelerates pubertal onset by more than 3 months in both boys and girls. Post-bedtime LAN was the most potent driver, suggesting that light suppresses melatonin during critical developmental windows, triggering premature HPG axis activation. This effect provides a developmental origin story for adult PCOS: early-life LAN exposure may permanently alter circadian set points, predisposing to later reproductive-endocrine dysfunction.

REV-ERBs (REV-ERBα/β, nuclear receptors acting as transcriptional repressors of BMAL1 and core circadian clock components), mitochondrial dysfunction, and alterations in ovarian clocks may represent other principal factors underlying melatonin dysregulation. The ovarian granulosa cell (GC) is not merely a passive target but an active circadian oscillator. Sun et al. (2021) [85] demonstrated that REV-ERBα/β downregulation in PCOS granulosa cells impairs mitochondrial biogenesis via the PGC-1α-NRF1-TFAM axis. PGC-1α (peroxisome proliferator-activated receptor gamma coactivator 1-alpha, transcriptional coactivator induced by exercise/cold/fasting), NRF1 (nuclear respiratory factor 1, transcription factor regulating nuclear respiratory chain genes), and TFAM (mitochondrial transcription factor A, which packages mtDNA nucleoids and drives mtDNA transcription/replication) ultimately increase autophagy and apoptosis. Crucially, pharmacological activation of REV-ERBs with SR9009 agonist rescued follicular development in a PCOS mouse model, directly linking circadian nuclear receptors to ovarian function. Similarly, Wang et al. (2021) [144] identified loss of clock transcription factor binding motifs in ATAC-seq peaks of PCOS granulosa cells, alongside dysregulated rhythmicity of corticotropin-releasing hormone (CRH), adrenocorticotropic hormone (ACTH), prolactin (PRL), and thyroid-stimulating hormone (TSH), indicating central-peripheral circadian decoupling. This decoupling may explain the frequent co-occurrence of PCOS with thyroid dysfunction and hypothalamic–pituitary–adrenal axis hyperactivity.

Notably, Kroneld et al. (2024) [161] found no association between PCOS and self-reported or sMEQ-derived chronotypes. It underscores a notable insight: subjective chronotype assessments can be unreliable proxies for true circadian phase or light exposure as chronotype is dynamic, influenced by age, social constraints, and, most critically, light history [29,162,163]. Objective actigraphy and light dosimetry are therefore essential.

### 2.4. Circadian Disruption and Endometriosis

The rising global prevalence of endometriosis [164] is responsible for 26% of infertility and cannot be fully explained by genetic or hormonal factors alone [165]. Growing evidence implicates circadian disruption and compromised light hygiene as a prominent environmental contributor [166,167,168]. A systematic review of 2573 patients found that 70.8% of women with endometriosis suffer from sleep disturbance, with prevalence rising sharply since 2018 and being significantly higher in China than in Europe or Iran [169]. Night shift work increases endometriosis risk by up to 98% when more than 50% of shifts occur at night, and women under age 35 working nights are 1.34 times more likely to receive an endometriosis diagnosis during fertility treatment [170,171]. These effects are mechanistically tied to LAN-induced melatonin suppression, which removes a critical antioxidant and anti-inflammatory shield: melatonin inhibits ectopic endometrial cell proliferation, reduces oxidative stress, and enhances uterine receptivity [172,173]. Genetic susceptibility further amplifies risk since genetic variants in core clock genes or melatonin receptors amplify vulnerability to CLH. Given the shared pathophysiology between PCOS and endometriosis (chronic inflammation, insulin resistance, anovulation), the HPG axis is likely to be disturbed also in relation to endometriosis progression. Notably, circadian disruption also dysregulates ferroptosis, a regulated iron-dependent cell death pathway, by suppressing BMAL1, leading to iron accumulation and enhanced survival of endometriotic lesions resistant to oxidative damage [174,175]. Notably, not only hazards of LAN, but diminished daylight can add to the interplay between endometriosis and CLH: higher residential UV exposure in early life was found to be protective [176], suggesting that natural light entrainment, not UV dose alone, matters [177].

Unlike merits of melatonin supplementation in women with PCOS, this intervention is poorly studied in women with endometriosis [123,172]. The few therapeutic trials of melatonin supplementation in endometriosis patients demonstrated clinical benefits. Schwertner et al. (2013) [129] conducted an 8-week randomized clinical trial showing that compared to placebo, 10 mg of nightly melatonin reduced endometriosis-associated chronic pelvic pain by 39.8% (95% CI: 12.88–43.01%), dysmenorrhea by 38.0% (95% CI: 15.96–49.15%), improved sleep quality, and decreased analgesic use by 80%. Mechanistically, melatonin blocked estradiol-induced epithelial–mesenchymal transition, invasion, and proliferation in endometriotic cells via Notch1/Numb upregulation [178]. Mosher et al. (2019) [179] confirmed melatonin receptor expression in eutopic/ectopic endometrium and demonstrated melatonin’s inhibition of estradiol-induced epithelial proliferation. No dedicated trials of light therapy for endometriosis were identified. Collectively, poor CLH emerges as a modifiable, population-level risk factor for endometriosis, acting through melatonin loss, clock gene dysregulation, and ferroptosis resistance, highlighting the potential for light-based behavioral and chronotherapeutic interventions.

### 2.5. Male Fertility

The global prevalence of male infertility reached approximately 56.5 million by 2019, reflecting a 76.9% increase in total cases and a 19% rise in standardized prevalence since 1990 [180]. This escalation exceeds projections based solely on traditional lifestyle factors or environmental toxins. Emerging evidence suggests that circadian dysregulation, driven largely by altered light-exposure patterns, may be a critically underappreciated driver of impaired spermatogenesis [181]. The concurrent rise in evening blue-enriched screen exposure over recent decades provides a compelling candidate mechanism. However, existing research currently lacks quantitative metrics for this exposure in melanopic Equivalent Daylight Illuminance (m-EDI). Although Gimenez et al. (2022) [15] provide a tool to estimate m-EDI dose (melanopic lux hours) as a predictor of melatonin suppression, establishing standardized m-EDI quantification for screen use remains a vital priority for future research.

Animal studies showed that circadian regulation of male fertility is mediated by core clock genes that govern testicular function at multiple levels [182]. Alvarez et al. (2008) [183] first demonstrated that BMAL1 knockout mice are infertile due to impaired Leydig cell steroidogenesis, with reduced StAR (Steroidogenic Acute Regulatory protein, a rate-limiting mitochondrial cholesterol transport protein that delivers cholesterol from the outer to the inner mitochondrial membrane) expression and low testosterone, establishing BMAL1 as a direct transcriptional regulator of androgen production. Liang et al. (2013) [184] extended this link to core clock genes by showing that testis-specific CLOCK knockdown reduces sperm acrosin activity and blastula formation, revealing a novel, non-systemic role for CLOCK in spermatid function. Li C. et al. (2018) [185] further identified CRY1 as essential for suppressing germ cell apoptosis and maintaining sperm count, with dysregulated miRNAs targeting core clock components, suggesting a feedback loop critical for testicular function. Moustafa (2020) [86] revealed that chronic circadian misalignment via prolonged light or dark alters hypothalamic and testicular clock gene expression (PER1-2, CRY1-2, BMAL1, CLOCK, REV-ERBα), elevates reproductive hormones as compensatory responses, and enhances antioxidant defenses, indicating adaptive plasticity. Ogo et al. (2021) [186] demonstrated that developmental exposure to constant light during gestation permanently impairs testicular structure, Sertoli cell number, and adult testosterone and sperm output in male offspring, highlighting a critical window of vulnerability. Travicic et al. (2025) [187] confirmed that light deprivation during puberty disrupts Leydig cell maturation, delaying the androgen-dependent transition from round to elongated spermatids and reducing sperm function, a phenomenon relevant to blind individuals and populations in polar regions. Liu G. et al. (2026) [188] showed that dynamic circadian instability (LDDL cycle) induces severe testicular oxidative stress (↑ NOX4/5 [NADPH oxidases 4/5, superoxide generators]; ↓ HO-1 [heme oxygenase-1, antioxidant enzyme]/SOD [superoxide dismutase]), germ cell apoptosis (↑ Bax/Bcl-2 ratio [pro- vs. anti-apoptotic proteins]; ↑ caspase-3 [executioner protease]), and testosterone decline, with effects worse than fixed shift work, implicating rhythm instability, not just phase shift, as a key driver of damage.

In humans, rotating shift work is associated with a 26% higher risk of low sperm count (defined as less than 120.10^6^ sperm/mL [180]). This finding has been observed in both undergraduate students and mice, where sperm count recovered following circadian rhythm realignment. Furthermore, the downregulation of meiotic genes, specifically those in the homologous recombination pathway, suggests a link between desynchrony and genomic instability in spermatocytes [189]. Night shift work also independently increases oxidative stress in semen. Importantly, a 3-month course of antioxidant therapy led to a four-fold greater reduction in reactive oxygen metabolites and more significant improvements in semen quality among night workers compared to day workers, suggesting they may experience a disproportionately larger fertility benefit from antioxidant supplementation [190]. In a study of 50 patients with idiopathic oligoasthenoteratozoospermia (iOAT, characterized by low sperm count, motility, and abnormal morphology) who were exposed to LAN, serum and seminal melatonin values were lower (*p* < 0.0001). A significant correlation was also found between lower sperm motility and reduced serum melatonin (r = 0.614, *p* < 0.0001) [191]. Additionally, Green et al. (2020) [192] found that more than 1-h daily self-reported screen exposure in the evening was associated with reduced sperm concentration and motility in an observational cohort of 116 participants (r = −0.392 for motility). This association persisted independently of sleep duration, implicating direct signaling pathways from the retina’s photoreceptors. Tian et al. (2024) [193] (observational cohort, n = 1991 donors) provided population-level evidence of chronic outdoor LAN ≥ 100 nW/cm^2^/sr linked to dose-dependent declines in sperm motility: VCL (actual swimming speed) ↓ 5.5 µm/s, LIN (straightness of movement) ↓ 0.043, NP (movement without forward progress) ↑ 3.9% per 100-unit increase, indicating slower, less efficient sperm. These effects were most pronounced in men under 25, likely due to heightened sensitivity to circadian disruption during reproductive development [193]. Lu et al. (2024) [194] used Mendelian randomization to show that genetically predicted evening chronotype was causally associated with lower bioavailable testosterone, but not directly with infertility, suggesting that hormonal modulation precedes functional decline. Zhou et al. (2025) [160] confirmed that poor sleep quality, independently of hormones, reduces sperm concentration and motility and halves pregnancy success in infertile couples. However, the bidirectionality of the relationship should be considered, since infertility induces stress, which subsequently disrupts sleep. Consequently, while CLH may contribute to poor sleep quality and diminished sperm parameters, reverse causality and the stress-infertility-sleep loop constitute significant confounding factors in observational studies. Furthermore, we caution against conflating pharmacological melatonin supplementation (e.g., implants in rams, oral tablets in humans) with endogenous melatonin secretion, as these interventions have distinct pharmacokinetics and physiological timing. Light hygiene aims to restore natural rhythms, not to replace them with exogenous supplements, and these distinct mechanisms should not be used interchangeably when interpreting therapeutic effects. Therefore, melatonin formulations specifically designed for this purpose would be needed for effective therapeutic applications.

Recent studies provided some mechanistic insights to the interplay between circadian mechanisms and male fertility, revealing that Sertoli cell-specific clock machinery drives the rhythmic production of retinoic acid, thereby coordinating both spermatogonial differentiation (via Zbtb16a) and subsequent gamete fusion potential (via Izumo1) [195]. Pavlovic et al. (2022) [196] and Marinkovic et al. (2021) [197] detailed how circadian disruption in Leydig cells suppresses steroidogenic genes (Star, Cyp11a1, Hsd3b), impairs mitochondrial dynamics (fusion ↓, mitophagy ↑), and reduces ATP production, creating an energy crisis that underpins testosterone deficiency. Xie et al. (2018) [198] and Pakmanesh et al. (2024) [199] demonstrated circannual rhythms in human semen: sperm concentration and count peak in spring, morphology in summer, and motility in spring, reinforcing that seasonal changes in photoperiod and light exposure patterns are biologically embedded regulators. Shimomura et al. (2020) [200] observed that fertile men reach their highest total motile sperm count in the evening, whereas infertile men show no 24-h variation. Interestingly, accumulated light exposure is the environmental variable that most strongly predicts the evening rise of REV-ERBα transcripts in human blood mononuclear cells [201]. In that cohort, maximum correlation with light occurred at 19:31 ± 30 min on long light days, mirroring the timing of the sperm motility maximum. Whether testicular REV-ERBα tracks the same light-driven evening profile, and whether its amplitude loss parallels the flat sperm curves seen in infertile men remains to be tested. Recent reviews [62,202] synthesized that circadian disruption is not merely a correlate but a mechanistic driver of male infertility, acting through clock gene dysregulation, oxidative stress, mitochondrial failure, and loss of temporal coordination in spermatogenesis and steroidogenesis, positioning CLH as a modifiable, non-pharmacological target for improving global male reproductive health. Key human studies are summarized in Table 3.

Melatonin, a circadian-regulated neurohormone, is emerging as a central node linking CLH to male fertility [203,204,205,206,207]. Across vertebrate species, seminal melatonin concentrations rise in dark phases and decline under light pollution; yet at temperate-to-arctic latitudes, a true 12L:12D photoperiod is encountered only fleetingly around the equinoxes, so seasonal melatonin rhythms are especially relevant [208]. Exogenous implants that mimic the long nocturnal peaks of winter reproducibly rescue sperm compromised by short photoperiod, heat stress or metabolic syndrome in diurnal animals. In rams, 54 mg sub-cutaneous implants elevate seminal melatonin about 3-fold within 90 days, doubling in vitro blastocyst yield and shifting seminal metabolome toward glutamate, carnitine and arginine—metabolic signatures of enhanced mitochondrial competence [209,210]. Similar protocols reduced DNA fragmentation and post-thaw motility loss in cryopreserved Merino sperm [211], while mitigating heat-stress-induced morphological anomalies via improved testicular perfusion [212]. Beyond seasonal breeders, human sperm cryopreserved with 0.01 mM melatonin retain higher motility and lower MDA (malondialdehyde, a marker of oxidative stress) and ROS (reactive oxygen species) [213]. A meta-analysis of rodent metabolic-disease models shows melatonin uniformly restores sperm count, motility and Johnsen score while normalizing testosterone, low-density lipoprotein cholesterol, and testicular glutathione peroxidase/superoxide dismutase activity [214]. These benefits extend to pollutant-induced infertility: melatonin reverses testicular endoplasmic reticulum stress and apoptosis provoked by DEHP (di(2-ethylhexyl) phthalate), cadmium, arsenic and 18 other toxicants, up-regulating DDX3Y (DEAD-box helicase 3, Y-linked) and other spermatogenic hub genes [215,216,217]. Collectively, the data position timed melatonin supplementation, aligned with individual circadian phase and the extreme annual photoperiod swings at higher latitudes as a practical CLH intervention to curtail the ongoing male-infertility crisis.

## 3. Maternal CLH–Metabolic Axis in Fertility

A substantial and growing body of evidence now demonstrates that maternal exposure to unnatural light–dark cycles, particularly LAN, disrupts fetal developmental programming by inducing chronodisruption of maternal circadian signaling. This disruption has cascading consequences for metabolic homeostasis, fetal growth trajectories, and increased lifelong risk of metabolic and neurodevelopmental disorders in offspring [218,219,220]. Crucially, these effects converge on the kisspeptin (KP) system [87,221]: hypothalamic KP neurons, which sit anatomically and functionally between the SCN clock and the GnRH pulse generator, integrate circadian, metabolic, and sex-steroid signals to time the pre-ovulatory gonadotropin surge [222,223]. The pathway linking LAN → melatonin suppression → RFRP-3 → GnRH desynchronization involves steps that have been experimentally demonstrated in both animal models and humans, though key distinctions exist. In animal models, LAN suppresses nocturnal melatonin, which in turn modulates RFRP-3 (RFamide-related peptide-3, a gonadotropin-inhibitory neuropeptide) activity. While RFRP-3 is generally considered an inhibitor of GnRH, the precise mechanism by which melatonin suppresses RFRP-3 activity remains unclear. Some evidence suggests melatonin may act directly on RFRP-3 neurons or indirectly through SCN pathways [224]. In humans, epidemiological studies correlate LAN with disrupted melatonin rhythms and reproductive outcomes, but direct mechanistic studies on RFRP-3 modulation are lacking. Thus, the “removal of the brake” hypothesis is primarily inferred from animal data, with human studies providing support from relational evidence.

Grounded in the foundational observation that the in utero environment is inherently rhythmic [225], and that the fetal circadian system is entrained not by light directly but by maternal hormonal cues, most critically melatonin [226,227,228], chronodisruption during pregnancy represents a pervasive, modifiable environmental insult. Fetal entrainment by maternal melatonin is particularly dominant during early to mid-gestation, when the fetal SCN is not yet responsive to light [226]. KP neurons themselves express molecular clock genes (CLOCK, BMAL1, PER2), and their transcriptional rhythms are phase-locked to maternal melatonin; hence, LAN-induced clock-phase shifts in KP circuitry can permanently mis-set the offspring’s metabolic-reproductive pacemaker [222].

Human epidemiological and experimental animal data converge to demonstrate the effects of LAN, but the distinction between these lines of evidence is critical. In animal models, LAN suppresses the maternal melatonin rhythm, desynchronizes peripheral clocks, and alters cortisol, vasopressin, and thyroid hormone dynamics, thereby impairing fetal metabolic and neuroendocrine development. In humans, epidemiological studies link LAN to adverse pregnancy outcomes, but direct mechanistic studies are limited. In parallel, placental KP expression follows a circadian pattern that is melatonin-responsive; LAN flattens this placental KP rhythm, reducing nutrient-signaling efficiency and contributing to fetal macrosomia independently of maternal glycemia [221]. Critically, these disruptions occur not only during shift work [218,229] but also through ubiquitous LAN in urban populations. In sheep, simulated shift work during early gestation reduced lamb birth weight and impaired glucose tolerance, despite maternal metabolic adaptation later in pregnancy, highlighting the vulnerability of early fetal development [229]. Rodent models confirm that LAN during gestation abolishes neonatal melatonin rhythms, reduces corticosterone amplitude, and disrupts glucose and cholesterol homeostasis in offspring [230], effects reversible by exogenous melatonin [226], underscoring its role as the primary synchronizer for the fetal adrenal.

Human studies extend these findings to clinical outcomes, though causality remains inferential. Elevated LAN exposure during the first and second trimesters is associated with increased risk of preterm birth (PTB) [231], gestational diabetes mellitus (GDM) [232,233]), and macrosomia [234], with dose–response and nonlinear thresholds identified by restricted cubic spline analysis [232,233]. Notably, LAN correlates with elevated fasting glucose, insulin, and HOMA-IR (Homeostatic Model Assessment of Insulin Resistance) in early pregnancy, even before GDM diagnosis [235], suggesting a direct effect on glucose dysregulation. Furthermore, maternal LAN (10 p.m.–1 a.m.) is independently associated with increased depression and stress in the third trimester [236], while poor sleep quality and short sleep duration, both linked to LAN, elevate offspring risk of congenital heart disease [237,238]. Current hypotheses suggest that LAN may contribute to congenital heart disease through sleep disruption, though direct mechanistic studies are lacking. In a nationwide cohort of 717,113 Austrian births, higher LAN was associated with dose-dependent increases in prolonged labor (medium LAN: OR 1.22, 95% CI: 1.14–1.31; high LAN: OR 1.43, 95% CI: 1.30–1.57; both *p* < 0.0001) and adverse neonatal outcomes (medium LAN: OR 1.07, 95% CI: 1.04–1.10; high LAN: OR 1.12, 95% CI: 1.07–1.16; both *p* < 0.0001), including a modest but significant rise in very preterm delivery (<28 weeks; *p* = 0.04), directly linking LAN to labor dystocia and neonatal morbidity [239].

Emerging mechanisms involve gut microbiota remodeling, though human data are limited. Maternal LAN exposure alters maternal gut microbial composition (e.g., reduced Prevotella_2, increased Coprococcus_3) in animal models [240], but whether these changes occur in human cohorts or whether causality has been tested (e.g., via microbiota transfer) remains unclear. Additionally, light timing is critical: a U-shaped relationship between lights-out time and nocturnal SpO_2_, with a turning point at ~10 p.m., demonstrates that both late and very early bedtimes impair oxygenation, particularly in older and obese mothers [241]. Chrononutrition is intertwined: nighttime eating (9 p.m.–6 a.m.) independently increases PTB risk (OR 5.7) [242], suggesting that LAN promotes misaligned feeding behaviors that compound metabolic stress.

In this context, it is crucial that circadian amplitude and phase, not merely light intensity or photoperiod, are predictive of metabolic disease risk [29,30]. In the UK Biobank, brighter nights and smaller circadian amplitude independently increased type 2 diabetes risk (HR up to 1.53) [243], with effects comparable to moderate genetic risk. Novel actigraphy-derived indices, such as nocturnal excess blue light (NEIbl) and daylight deficit index (DDIbl), quantify circadian-disruptive light exposure. NEIbl measures excessive blue-light exposure during the night, while DDIbl assesses insufficient daylight exposure during the day [157]. These indices correlate more strongly with BMI and cortisol than traditional sleep metrics, particularly in carriers of the MTNR1B rs10830963 G-allele [157], revealing gene-environment interactions in metabolic susceptibility. Critically, this MTNR1B polymorphism, known to impair melatonin signaling and alter insulin secretion [155,244], likely heightens maternal susceptibility to circadian metabolic disruption from poor CLH during pregnancy, potentially exacerbating risks of gestational glucose intolerance, fetal overgrowth, and long-term offspring metabolic disease, a hypothesis yet to be formally tested in pregnant cohorts. This polymorphism may act as a genetic amplifier of CLH disruption, rendering the maternal–fetal axis hypersensitive to LAN. Conversely, natural daylight exposure emerges as a potent countermeasure: in individuals with T2DM, daylight exposure improved glucose control, increased fat oxidation, and advanced circadian phase in muscle [245], suggesting that dynamic light interventions, particularly those enhancing the circadian amplitude, are essential components of modern prenatal care.

Beyond melatonin receptor genes, circadian clock genes and their particular polymorphic variants deserve attention. Hodžić et al. (2018) [81] identified CLOCK rs6850524-G and rs11932595-G as significant risk alleles for idiopathic recurrent spontaneous abortion (IRSA), with ORs of 2.28 and 1.47, respectively. These same variants were associated with male infertility [82], suggesting a pleiotropic reproductive impact. At the molecular level, the lncRNA (long non-coding RNA) TCONS_00265853–miR-421-5p–CLOCK axis provides a mechanistic bridge [246]: CLH downregulates TCONS_00265853, upregulates miR-421-5p, and suppresses CLOCK, leading to MAPK hyperactivation, ovarian apoptosis, and PCOS-like phenotypes. Aci et al. (2023) [247] found that infertile women had significantly higher CLOCK protein expression and higher frequency of the PER3 DD (4/4) genotype, which correlates with blunted melatonin rhythms and elevated LH/prolactin, a hormonal profile consistent with anovulation and endometriosis.

Nuclear receptor subfamily 1 group D member 1 (REV-ERBα) and its paralog REV-ERBβ are core circadian clock components that integrate metabolic and circadian systems. Within the SCN, REV-ERBs act as molecular conduits, translating timing signals into coordinated neuroendocrine rhythms [83,248]. The link between REV-ERBα activity and light is evident in humans, where REV-ERBα expression in blood mononuclear cells shows strong seasonality, peaking in summer and reaching a nadir in winter, with light exposure being a predictor of REV-ERBα morning expression [201]. Furthermore, NR1D1 (REV-ERBα) senses heme as a central regulator of erythropoiesis, directly linking circadian-light hygiene to hemoglobin synthesis [249,250]. REV-ERBs synchronize peripheral physiology with the central pacemaker. For instance, their expression in SCN GABAergic neurons is essential for generating the 24-h rhythm of hepatic insulin sensitivity [251]. This alignment is crucial, as a dissonance between environmental conditions and endogenous circadian rhythms causes metabolic disruption [252]. Such desynchronization, often from mistimed light or food intake, uncouples peripheral clocks from the SCN and promotes systemic disorders, including reproductive dysfunction [253]. This circadian–metabolic link is exemplified in PCOS, where REV-ERB expression is significantly reduced in granulosa cells [85]. Crucially, REV-ERBα acts as a central regulator of mitochondrial quality in granulosa cells by inhibiting excessive mitophagy and promoting biogenesis. While REV-ERB agonists (e.g., SR9009) rescued mitochondrial function and folliculogenesis in PCOS mouse models [85,88], human pharmacological studies remain limited to Phase I safety trials without reproductive endpoints. The role of REV-ERBs extends to other reproductive processes. Disruption of REV-ERBα can alter gonadotropin-releasing hormone (GnRH) pulsatility, affecting downstream LH and FSH secretion [87,89]. Furthermore, in an inflammatory model of miscarriage, decreased REV-ERBα expression was associated with a pro-inflammatory bias in decidual macrophages, and its pharmacological activation restored immune balance and protected pregnancy [254]. The expression of other core oscillators, like Neuronal PAS Domain Protein 2 (NPAS2), has also been demonstrated in PCOS, and their restoration improves outcomes [145]. Additionally, light mediation of REV-ERBα rhythm can be clinically relevant for mood-oriented strategies, as higher morning expression independently predicts lower depression scores in Arctic residents [255], a known co-factor of fertility [256,257,258,259], which will be addressed in detail in the next section.

Furthermore, the effect of light exposure on hemoglobin indices merits consideration, particularly given its association with improved live birth rates following iron repletion in cases of iron deficiency [260]. Iron deficiency itself is a common, modifiable contributor to unexplained infertility and adverse pregnancy outcomes [261]. Obianeli et al. (2024) [262] highlight that anemia affects up to 30% of antenatal women and is associated with low birth weight, preterm birth, stillbirth, and neurocognitive deficits of the newborns. Although oral iron raises hemoglobin, a substantial proportion (about 45 to 50%) of women fail to respond to iron supplementation due to poor adherence and side effects, prompting increased use of intravenous iron. Emerging evidence suggests that circadian-aligned light exposure can enhance key hematological indices such as hemoglobin and mean corpuscular hemoglobin, potentially improving iron absorption and erythropoiesis [263].

Circadian regulation also extends to later stages of reproduction, including implantation, placentation, fetal development, and parturition. The placenta is not merely a passive conduit but a hormonally active tissue with circadian gene expression and responsiveness to maternal melatonin and glucocorticoid signals, which may help coordinate nutrient transfer, oxidative defense, and timing of fetal developmental programs [264,265,266,267]. Experimental and review data indicate that maternal melatonin supports placental integrity by limiting oxidative stress, modulating trophoblast function, and helping maintain a synchronized maternal–placental–fetal rhythm, whereas artificial LAN during pregnancy is associated with disrupted maternal circadian signaling and adverse placental and neonatal outcomes. Although direct evidence linking CLH to placental health remains more limited than the evidence for ovulatory or semen-related endpoints, the available literature supports placentation as a biologically plausible downstream target of circadian disruption. Taken together, CLH may influence reproduction across the full trajectory from menstrual regularity and gamete quality to implantation, gestation, placental function, and birth outcomes.

In conclusion, the CLH–metabolic axis is robustly supported by converging evidence from mechanistic insights, summarized in Table 4. Evidence includes human epidemiological data linking LAN to adverse pregnancy outcomes (e.g., preterm birth, gestational diabetes, macrosomia), with demonstrable dose–response relationships. Animal models further elucidate melatonin-mediated entrainment of fetal circadian rhythms, especially during early to mid-gestation. Molecular evidence highlights REV-ERBα’s crucial role in integrating circadian and metabolic signals, with therapeutic implications for PCOS. Gene-environment interactions, such as MTNR1B polymorphisms, amplify the susceptibility to LAN-induced disruption. Finally, interventional studies indicate that circadian-aligned light exposure may improve hematological indices. Collectively, maternal circadian disruption from LAN emerges as a dose- and time-sensitive, multi-system risk factor for adverse pregnancy outcomes and offspring metabolic programming, with a pronounced peak vulnerability in early gestation. This risk is modulated by sleep, nutrition, microbiota, and genetic factors. Therefore, CLH-supporting interventions, including reducing nocturnal light exposure, increasing daylight exposure, and supporting melatonin/kisspeptin rhythms promise significant public health benefits for fertility and intergenerational metabolic health.

## 4. CLH-Mood Axis: Depression, Anxiety, and Reproductive Decision-Making

CLH disruptions profoundly influence mood regulation, with poor CLH, both LAN [268,269] or insufficient morning daylight [269] exacerbating depression and anxiety symptoms that cascade into impaired reproductive decision-making and fertility outcomes [236,270,271]. This proposed CLH–mood–fertility link is supported by a mix of human epidemiology and animal mechanistic studies, but the full causal chain has not been demonstrated end-to-end in humans. These effects manifest across preconception, gestational, and postpartum phases, particularly in women vulnerable to seasonal light deficits at high latitudes. In pregnant women, LAN from 10 p.m. to 1 a.m. correlates with elevated stress (*β* = 0.212, *p* = 0.037) and depression (*β* = 0.228, *p* = 0.024) in the third trimester, alongside poor sleep quality predicting anxiety *(β* = 0.243, *p* = 0.002) and depression (*β* = 0.259, *p* = 0.001) earlier in gestation [236]. The 10 p.m.–1 a.m. interval likely reflects a biologically vulnerable nocturnal window coinciding with the time frame of highest metabolic vulnerability [157]. However, whether this window can be formally established as a circadian phase-sensitivity window should be examined in future studies. Animal models confirm that dim LAN during pregnancy and postpartum reduces serotonin (5-HT) and brain-derived neurotrophic factor (BDNF), inducing depression-like behaviors (e.g., reduced sucrose preference, increased immobility) via disrupted hippocampal PER1 expression and circadian rest-activity rhythms [271]. However, the light intensities used in these animal studies are typically experimental dim-light conditions rather than direct equivalents of domestic lighting or screen-derived light, so clinical translation should be made cautiously. Morning melEDI above 50 is linked to fewer depressive and stress symptoms across medicated (Selective Serotonin Reuptake Inhibitor users) and non-medicated groups, underscoring the role of light timing in mood independently of chronotype or pharmacology [272]. Depression is further associated with excessive gestational weight gain (*β* = 0.239, *p* = 0.039), potentially compounding fertility challenges through metabolic dysregulation, though direct sunlight exposure shows no effect on sleep or weight [273]. Accordingly, the SAD–fertility argument is best framed as an indirect pathway mediated through circadian disruption, sleep, and mood rather than as a direct fertility effect.

Prevalence of latitude-dependent seasonal affective disorder (SAD) (pooled 5.01%, rising with northern exposure; *β* = 0.2, *p* < 0.001) amplifies these risks, with maternal fall-winter SAD history interacting with conception season to impair child neurodevelopment (e.g., cognitive flexibility in girls), suggesting intergenerational reproductive costs [274,275]. These associations are primarily observational, and the pathway to fertility is inferred through circadian and mood-related mechanisms rather than shown as a direct SAD-to-fertility chain. Perinatal depression cohorts reveal 12% prevalence tied to sleep-circadian deficits, amenable to bright light therapy (BLT) for prevention and treatment [276]. BLT has been studied in pregnant patients as an intervention, but its fertility-specific effects remain untested.

Modern lifestyle misalignment associated with chronically compromised CLH may couple mood disturbances to reproductive hormones through hypothalamic–pituitary–gonadal desynchrony, while population data link moderate-to-severe depression with infertility risk, with 6.57% of the association accounted for by lipid markers such as the Non-HDL Cholesterol to HDL Cholesterol Ratio [258,277]. In these analyses, BMI and related metabolic factors should be treated as potential confounders or intermediaries, because some studies suggest obesity-related pathways, whereas others point to a stronger direct effect. Taken together, CLH optimization through timed daylight exposure or bright light therapy emerges as a low-cost strategy that may help reduce depression-related delays in family planning and fecundity.

## 5. Future Outlook and Applications

To translate these mechanistic insights into actionable clinical guidance, we propose quantitative thresholds for circadian-light exposure based on recommendations from Brown et al. (2022) [9]. These thresholds balance standardization with acknowledged inter-individual variability [9,157,278,279,280,281]. (e.g., 50-fold difference in evening melatonin suppression sensitivity) [269], Table 5. These thresholds provide reproducible benchmarks for future CLH intervention studies while acknowledging that sensitivity varies among individuals. Clinicians should interpret these recommendations as dynamic targets rather than rigid cutoffs, adjusting them based on individual chronotype, genetic susceptibility (e.g., MTNR1B, CLOCK variants), and reproductive outcomes.

Overall, CLH aligns with the framework of Foster & Roenneberg’s [282] who documented humanity’s transition from ‘environmental coupling’ to ‘circadian misalignment.’ Daily and seasonal geophysical cycles historically regulated temporal biology across species, yet increasing isolation from these geophysical cycles through artificial lighting and 24/7 working practices has created a false sense of biological independence from ancestral rhythms. The circadian clock remains dominant in organizing 24-h physiology, with sunlight as the primary entraining cue, while social structures like alarm clocks have a limited effect on endogenous timing. Biology and society now appear in serious opposition, with documented health consequences. Despite industrialized isolation from changes in temperature, food availability, and photoperiod, seasons still influence birth timing and health. Current fertility trends may reflect a profound biological consequence of this isolation from ancestral rhythmic anchors, mediated by circadian-light misalignment.

## 6. Conclusions: Reclaiming Circadian Light for Human Continuity

Accumulating evidence suggests that environmental circadian disruption is an underrecognized contributor to reproductive dysfunction in both women and men. Across the studies reviewed here, poor Circadian-Light Hygiene is associated with altered melatonin signaling, clock-gene dysregulation, impaired neuroendocrine timing, and adverse reproductive outcomes, with urbanization and modern light exposure patterns likely intensifying these effects. Although much of the current evidence is observational or experimental rather than causal in humans, the convergence of mechanistic, epidemiological, and interventional data supports the view that circadian alignment is relevant to reproductive health.

Future research should focus on prospective longitudinal studies, standardized quantification of light exposure, and circadian biomarkers that capture both timing and amplitude. Particular attention should be given to distinguishing general population prevention from clinical intervention in infertile couples, as the appropriate light-based strategies may differ across these settings. If confirmed in well-designed trials, circadian medicine could become a low-cost adjunct to conventional fertility care and a practical target for reproductive health promotion.

## Figures and Tables

**Table 3:** Light-at-Night and Circadian Disruption Impair Human Sperm Motility and Count: Key Studies.

Study	Exposure Metric	Population	Key Findings (Sperm Effects)
Green et al. (2020) [192]	Evening + post-bedtime screen use >1 h (questionnaire, smartphone & tablet)	n = 116 fertility clinic	↓ Total motility (r = −0.39), ↓ Progressive motility (r = −0.32), ↓ Concentration (r = −0.17), ↑ Immotile sperm (r = +0.38).
Hassan et al. (2020) [191]	LAN (self-reported night-light exposure)	n = 50 iOAT + controls	iOAT: serum melatonin ½ of controls (*p* = 0.0004); LAN further ↓ both serum & seminal melatonin (*p* < 0.0001 & 0.02). Serum melatonin ↑ with motility (r = 0.614).
Tian et al. (2024) [193]	Outdoor ALAN ≥ 100 nW/cm^2^/sr	n = 1991 donors	↓ VCL 5.5 µm/s, ↓ LIN 0.043, ↑ NP 3.9%/100-unit ↑ ALAN: ↓ Motility −4.7%, ↓ LIN −0.043, ↓ VCL −5.5 µm s^−1^, ↑ Non-progressive motility +3.9%; strongest impairment in men < 25 y.
Liu K. et al. (2020) [189]	Rotating shifts Work-related (rotating vs. permanent shift)/Circadian mis-alignment (MCTQ social-jet-lag)	n = 1346 Chinese men; n = 796 undergraduates	Rotating shift: OR 1.26 (1.05–1.52) for low sperm count (<120.10^6^). Each 1 h social jet-lag: OR 1.16 (1.02–1.31). Reversible with realignment in a model.
Boeri et al. (2025) [190]	Night-shift work	n = 96 European men (18–45 y)	Baseline: ↑ D-ROMs (1.2-fold, *p* = 0.01); 74% vs. 24% above threshold.

Notes: All studies report correlational associations, except [189] showing partial reversibility with circadian realignment. LAN measured via satellite radiance (nW/cm^2^/sr); screen exposure self-reported. VCL = curvilinear velocity; LIN = linearity; NP = non-progressive motility; D-ROMS = derivatives of reactive oxygen metabolites.

**Table 4:** Key Circadian Clock Pathways in Ovarian and Reproductive Health.

Circadian Component	Light/CLH Influence	Downstream Impact on Ovarian/Reproductive Health
CLOCK/BMAL1	CLOCK suppression via miR-421-5p	MAPK hyper-activation → ovarian apoptosis [248] (animal model)
REV-ERBα/β	Seasonal light-linked expression; can be suppressed by LAN	Mitochondrial quality control in granulosa cells; mouse PCOS models [85,88] GnRH pulsatility regulation [87,89] Heme sensing → erythropoiesis/anemia prevention [249,250] (light sensitivity, link to human red blood cells/anemia markers: [263]) Hepatic insulin sensitivity rhythm; mouse SCN [251] Anti-inflammatory macrophage balance in decidua ([254] mouse miscarriage) Morning expression → lower depression [255] Arctic humans

Notes: CLH = circadian-light-hygiene interventions (e.g., dawn-simulating lamps, evening blue-light filtering) REV-ERBα is the predominant isoform in human granulosa cells.

**Table 5:** Standardized Circadian-Light Thresholds for Reproductive Health.

Metric	Target Baseline Threshold	Biological Mechanism	Reproducibility & Variations
Daytime Exposure	Dynamic, >250 lux Melanopic EDI * [9]	Enforces HPG axis synchronization; elevates daytime alertness	Minimum threshold required to counteract evening light disruption
Evening Exposure	<10 lux Melanopic EDI * (3 h before bed) [9]	Prevents melatonin suppression; protects clock-gene expression	Highly variable; 50% melatonin suppression ranges from 6–350 lux [279].
Sleep Environment	0 lux (no ambient light) [9,282]	Eliminates nocturnal endocrine and metabolic disruption	Crucial for preventing night-to-night sleep-timing irregularity [280,281].
Timing & Regularity	Consistent daily schedule (±30 min)	Anchors master pacemaker; stabilizes peripheral tissue clocks	Avoid circadian phase shifts as they may induce reproductive axis desynchronization

* Melanopic Equivalent Daylight Illuminance (EDI); measured in melanopic lux. Note that thresholds should be personalized based at least on genetics, age, and seasonal aspects of light sensitivity.

## Data Availability

Not Applicable.

## Acronym List

5-HT	Serotonin
AMPK/mTOR	AMP-activated Protein Kinase/Mechanistic Target of Rapamycin
ATAC-seq	Assay for Transposase-Accessible Chromatin using sequencing
BDNF	Brain-Derived Neurotrophic Factor
BLT	Bright Light Therapy
BMAL1	Brain and Muscle ARNT-Like 1
CLH	Circadian-Light Hygiene
CLOCK	Circadian Locomotor Output Cycles Kaput
CRY	Cryptochrome
CYP17A1	Cytochrome P450 17A1
DDI	Daylight Deficit Index for Light Exposure
DDX3Y	DEAD-Box Helicase 3 Y-Linked
DEHP	Di(2-ethylhexyl) phthalate
DLMO	Dim Light Melatonin Onset
D-ROMS	Derivatives of Reactive Oxygen Metabolites.
melEDI	Melanopic Equivalent Daylight Illuminance
FSH	Follicle-Stimulating Hormone
GDM	Gestational Diabetes Mellitus
GnRH	Gonadotropin-Releasing Hormone
GPx	Glutathione Peroxidase
HDL-C	High Density Lipids Cholesterol
HPG axis	Hypothalamic–Pituitary–Gonadal axis
HOMA-IR	Homeostatic Model Assessment of Insulin Resistance
ipRGCs	Intrinsically Photosensitive Retinal Ganglion Cells
KP	Kisspeptin
LAN	Light at Night
LH	Luteinizing Hormone
LDDL	Light–Dark–Dark–Light
MDA	Malondialdehyde
MTNR1A	Melatonin Receptor 1A
MTNR1B	Melatonin Receptor 1B
NEIbl	Nocturnal Excess Index for Blue Light Exposure
NPAS2	Neuronal PAS Domain Protein 2
PCOS	Polycystic Ovary Syndrome
PTB	Preterm Birth
qPCR	quantitative Polymerase Chain Reaction
REV-ERBα/β	Nuclear Receptor Subfamily 1 Group D Member 1/2
RFRP-3	RFamide-Related Peptide-3
ROS	Reactive Oxygen Species
SAD	Seasonal Affective Disorder
SCN	Suprachiasmatic Nucleus
SERT	Serotonin Transporter
SRD5A1/2	Steroid 5-alpha-Reductase Types 1 and 2
SOD	Superoxide Dismutase
T2DM	Type 2 Diabetes Mellitus
TC	Total Cholesterol
TFR	Total Fertility Rate
